# Supplementary material for: Selective vulnerability of inhibitory networks in multiple sclerosis
Source: Acta Neuropathol. 2021 Jan 15;141(3):415–29. doi: 10.1007/s00401-020-02258-z (PMC7882577; doi:10.1007/s00401-020-02258-z)
Supplement: Supplementary file 1 — (DOCX 6152 kb) [file 401_2020_2258_MOESM1_ESM.docx]

**Demyelination induces selective vulnerability of inhibitory networks in multiple sclerosis.**

**Lida Zoupi^1^, Sam A. Booker^2,6,7^, Dimitri Eigel^3^, Carsten Werner^3^, Peter C. Kind^2,6,7^, Tara L. Spires-Jones^2,4^, Ben Newland^3,5^ and Anna C. Williams^1*^**

1. Centre for Regenerative Medicine, Institute for Regeneration and Repair, University of Edinburgh, Edinburgh, EH16 4UU, UK.

2.Centre for Discovery Brain Sciences, University of Edinburgh, Edinburgh, EH8 9XD, UK.

3.Leibniz-Institut für Polymerforschung Dresden e.V., Max Bergmann Center of Biomaterials Dresden, Hohe Straße 6, D-01069 Dresden, Germany.

4.UK Dementia Research Institute, University of Edinburgh, Edinburgh, EH8 9JZ, UK.

5.School of Pharmacy and Pharmaceutical Sciences, Cardiff University, Cardiff CF10 3NB, UK.

6.Simons Initiative for the Developing Brain, University of Edinburgh, Edinburgh, EH8 9XD, UK.

7. Patrick Wild Centre for Autism Research, University of Edinburgh, Edinburgh, EH8 9XD, UK.

***Corresponding author: Anna C. Williams,** [**anna.williams@ed.ac.uk**](mailto:anna.williams@ed.ac.uk)**, tel: +44(0) 131 651 9500**

**Online Resource Material**


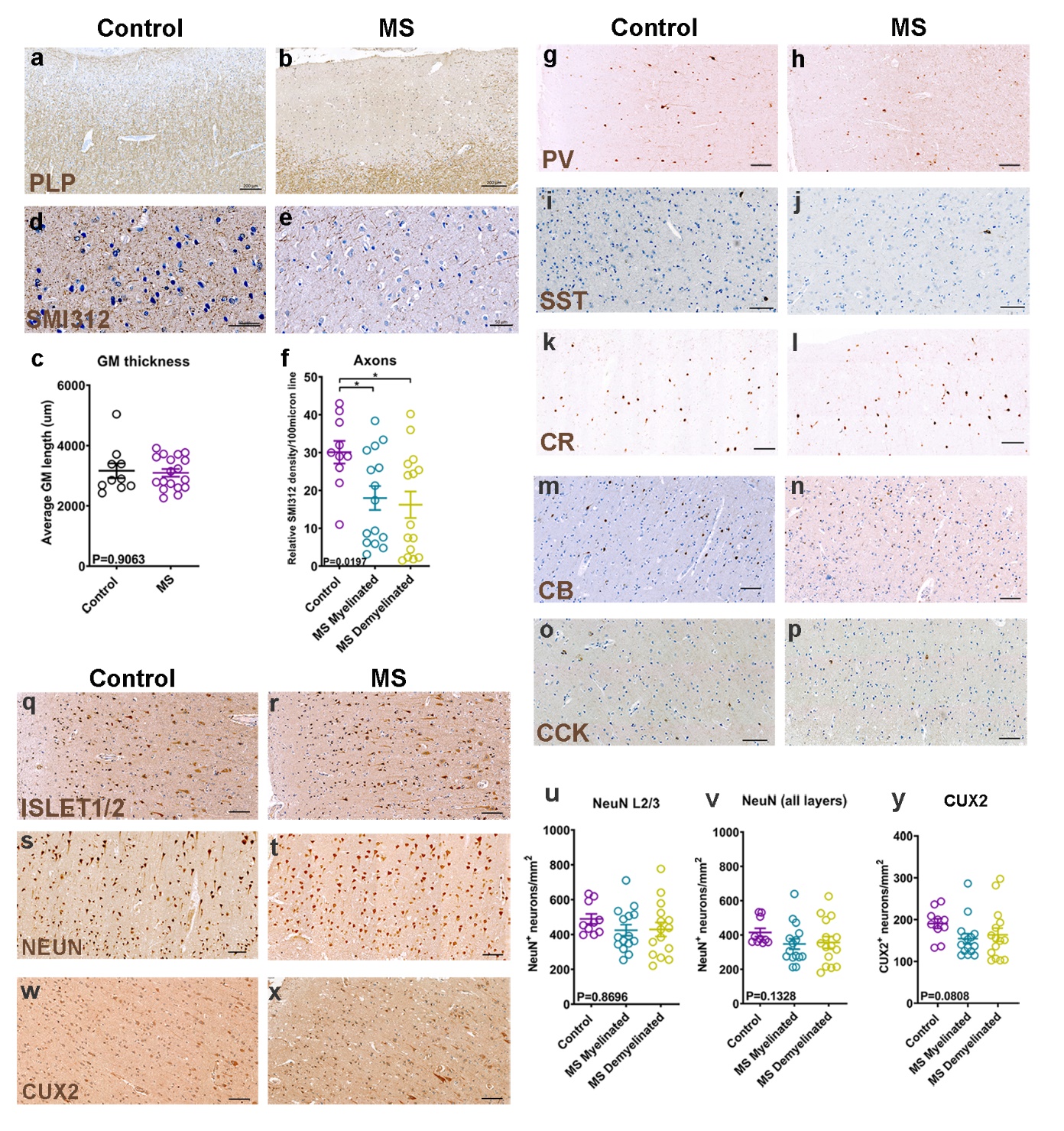


**Online Resource Fig. 1. a-b:** Immunohistochemistry of human control (a) and MS (b) motor cortex for the myelin marker PLP (brown) with hematoxylin counterstain (blue). Scale bar: 200μm. **c:** Grey matter (GM) thickness in control and MS motor cortex samples (Control: mean 3165 ± 245.3 SEM μm, N=10, MS: mean 3096 ± 126.8 SEM μm, N=18; each point is a patient, Mann-Whitney test). **d-e:** Immunohistochemistry of human control (d) and MS (e) motor cortex for the pan-axonal marker SMI312 (brown) with hematoxylin counterstain (blue). Scale bar: 50μm. **f:** Relative axonal (SMI312+) density crossing 100μm line in control and MS motor cortex for L2/3 (Control: 30.10± 2.983 SEM number of SMI312 axons/100μm, N=10, MS Myelinated: mean 17.99 ± 3.150 SEM number of axons/100μm, MS Demyelinated: mean 16.22 ± 3.499 SEM number of axons/100μm, N=15; each point is a patient, One-way ANOVA). **g-p:** Immunohistochemistry of human control and MS motor cortex for the interneuron markers PV (g,h), SST (i,j), CR (k,l), CB (m,n), CCK (o,p), all brown with hematoxylin counterstain (blue). Scale bars: 100μm. **q-r:** Immunohistochemistry of human control (q) and MS (r) motor cortex for the motor neuron marker ISLET1/2 (brown) with hematoxylin counterstain (blue). Scale bar: 100μm. **s-t:** Immunohistochemistry of human control (s) and MS (t) motor cortex for the pan-neuronal marker NEUN (brown). Scale bar: 100μm. **u:** Quantification of NEUN+ neuron density in control and MS motor cortex in L2/3 (Control: mean 489.6 ± 28.94 SEM neurons/mm^2^, N=10, MS Myelinated: mean 424.6 ± 31.95 SEM neurons/mm^2^, MS Demyelinated: mean 429.0 ± 40.35 SEM neurons/mm^2^, N=15; each point is a patient, One-way ANOVA). **v:** Quantification of NEUN+ neuron density in control and MS motor cortex in all six cortical layers (Control: mean 414.6 ± 24.16 SEM neurons/mm^2^, N=10, MS Myelinated: mean 347.8 ± 30.29 SEM neurons/mm^2^, MS Demyelinated: mean 356.9 ± 34.37 SEM neurons/mm^2^, N=15; each point is a patient, Kruskal-Wallis test). **w-x:** Immunohistochemistry of human control (w) and MS (x) motor cortex for the L2/3 projection neuron marker CUX2 (brown) with hematoxylin counterstain (blue). Scale bar: 100μm. **y:** Quantification of CUX2+ projection neuron density in control and MS motor cortex in L2/3 (Control: mean 190.6 ± 11.54 SEM neurons/mm^2^, N=10, MS Myelinated: mean 154.6 ± 11.86 SEM neurons/mm^2^, MS Demyelinated: mean 163.7± 15.82 SEM neurons/mm^2^, N=15; each point is a patient, Kruskal-Wallis test).


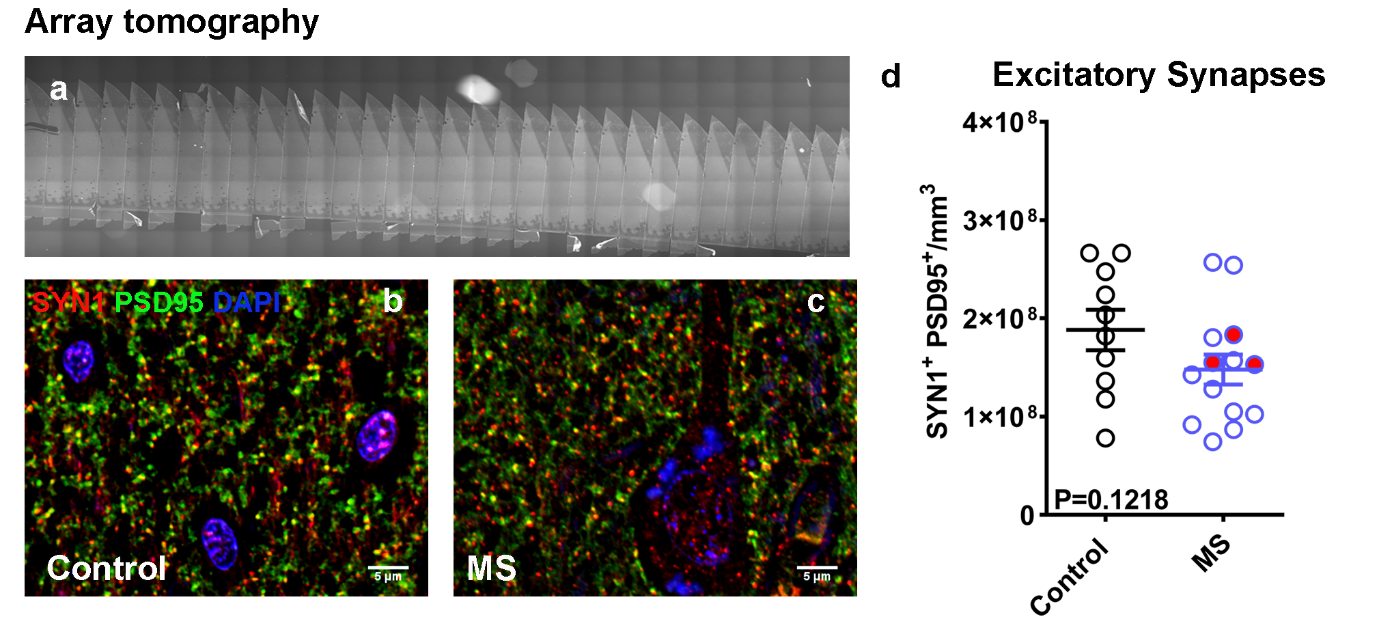


**Online Resource Fig.2. a:** Example of an array tomography (AT) ribbon (image taken with fluorescent microscope). Each ribbon consists of sequential 70nm-thick tissue sections. **b-c:** Representative images of AT sections from control (b) and MS (c) motor cortex, stained for SYN1 (red), PSD95 (green) with DAPI (blue). Scale bar: 5μm. **d:** Quantification of excitatory, SYN1+/PSD95+ synapses of control and MS motor cortex (AT samples, red-filled circles indicate demyelinated samples); (Control: mean 1.881e+008 ± 2.054e+007 SEM synapses/mm^3^, N=10, MS: mean 1.479e+008 ± 1.524e+007 SEM synapses/mm^3^, N=14; each point is a patient, unpaired t test).


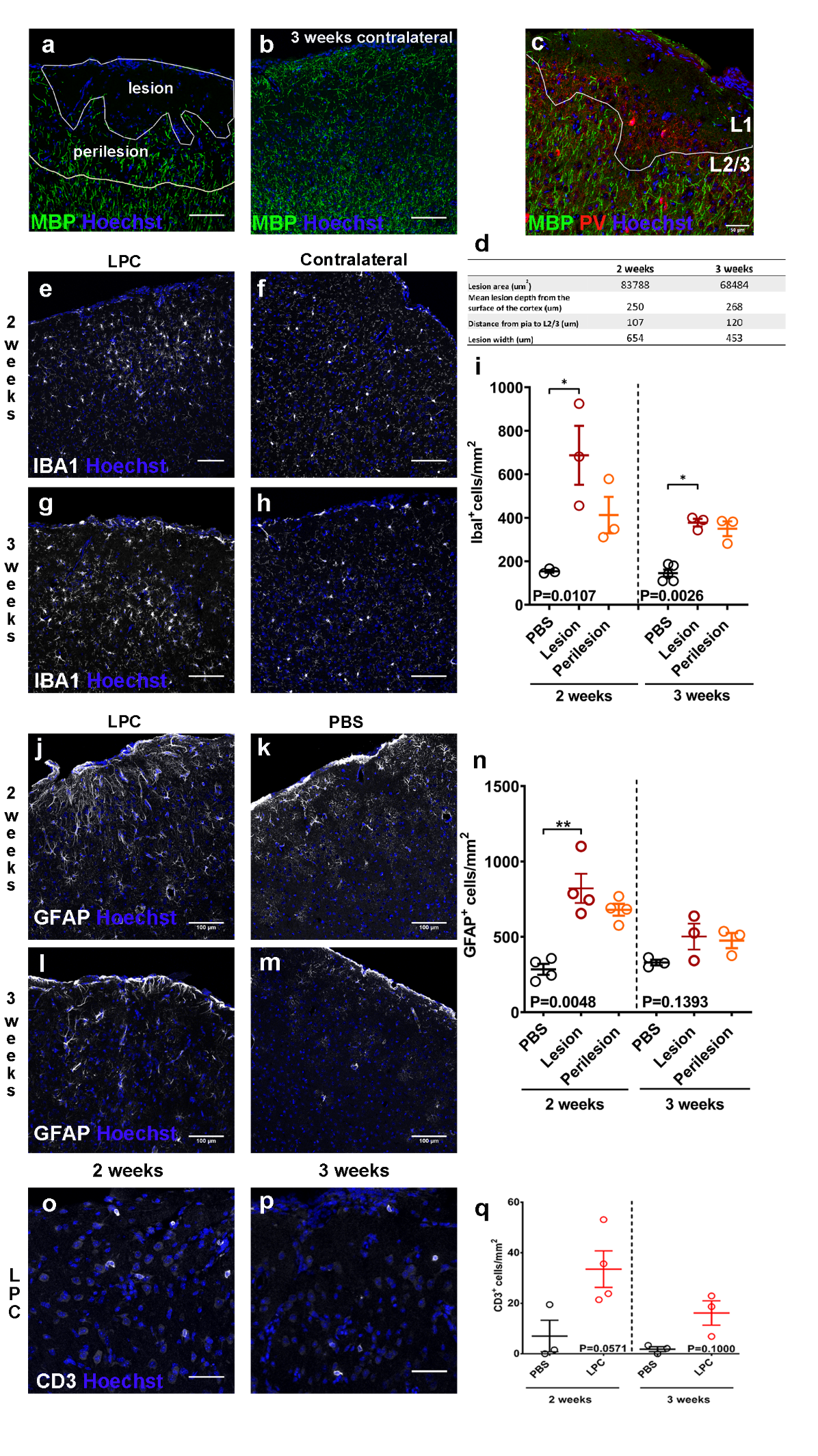


**Online Resource Fig.3. a:** Outline of a cortical lesion and perilesion areas after LPC-loaded cryogel placement onto the rodent cortex. Cortical coronal section stained for MBP (green) and Hoechst (blue). The area 150μm from the lesion border was considered as the perilesion area. Scale bar: 100μm. **b:** Immunohistochemistry for MBP (green) and Hoechst (blue) of the contralateral hemisphere of LPC-cryogel treated animal, 3 weeks post-surgery. Scale bar: 100μm. **c:** Cortical section of the lesion area of LPC-treated animals, 2 weeks post-surgery. Immunohistochemistry for MBP (green), PV (red) and Hoechst (blue). The layer1 (L1) and layers 2/3 (L2/3) are indicated and the lesion area is outlined with white line. The PV signal marks L2/3. Scale bar: 50μm. **d:** Table with mean lesion dimensions 2 and 3 weeks post-surgery. **e-h:** Immunohistochemistry of LPC-loaded cryogel animals for IBA1 (white) and Hoechst (blue) two (e) and three weeks (g) after cryogel placement. Contralateral hemisphere of the 2 week (f) and 3 week (h) after LPC-loaded cryogel placement. Scale bar: 100μm. **i:** Quantification of IBA1+ cell density at the upper cortical layers of PBS treated cortex (control), LPC-treated lesion and perilesion areas, for two (left) and three (right) weeks post–surgery (2 weeks PBS: mean 154.1 ± 6.606 SEM cells/mm^2^, N=3, Lesion: mean 687.3 ± 135.4 SEM cells/mm^2^, Perilesion: mean 412.6 ± 83.65 SEM cells/mm^2^, N=3; 3 weeks PBS: mean: 145.5 ± 16.66 SEM cells/mm^2^, N=5, Lesion: mean 377.7 ± 17.58 SEM cells/mm^2^, Perilesion: mean 349.6 ± 34.08 SEM cells/mm^2^, N=3; each point is an animal, Kruskal-Wallis test). **j-k:** Immunohistochemistry of PBS treated animals (k) and LPC treated (j) for GFAP (white) and Hoechst (blue) two weeks after cryogel placement. Scale bar: 100μm. **l-m:** Immunohistochemistry of PBS treated animals (m) and LPC treated (l) for GFAP (white) and Hoechst (blue) three weeks after cryogel placement. Scale bar: 100μm. **l:** Quantification of GFAP+ cell density in the upper cortical layers of PBS treated cortex (control) LPC-treated lesion and perilesion areas, two (left) and three (right) weeks post–treatment (2 weeks PBS: mean 284.9± 36.02 SEM cells/mm^2^, N=4, Lesion: mean 821.9 ± 96.71 SEM cells/mm^2^, Perilesion: mean 679.7 ± 39.53 SEM cells/mm^2^, N=4; 3 weeks PBS: mean 329.8 ± 18.18 cells/mm^2^, N=3, Lesion: mean 502.3 ± 86.19 SEM cells/mm^2^, Perilesion: mean 475.2 ± 50.20 SEM cells/mm^2^, N=3; each point is an animal, Kruskal-Wallis test). **o-p:** Immunohistochemistry of LPC treated animals two (o) and three (p) weeks after LPC-loaded cryogel placement for CD3 (white) and Hoechst (blue). In both cases the area of the lesion is shown. Scale bar: 50μm. **o:** Quantification of CD3+ cell density in the upper cortical layers of PBS treated cortex (control) LPC-treated areas, two (left) and three (right) weeks post–surgery (2 weeks PBS: mean 7.012 ± 6.246 SEM cells/mm^2^, N=3, LPC: mean 33.51 ± 7.246 SEM cells/mm^2^, N=4; 3 weeks PBS: mean 1.820 ± 0.9724 SEM cells/mm^2^, N=3, LPC: mean 16.15 ± 4.814 SEM cells/mm^2^, N=3; each point is an animal, Mann Whitney test).


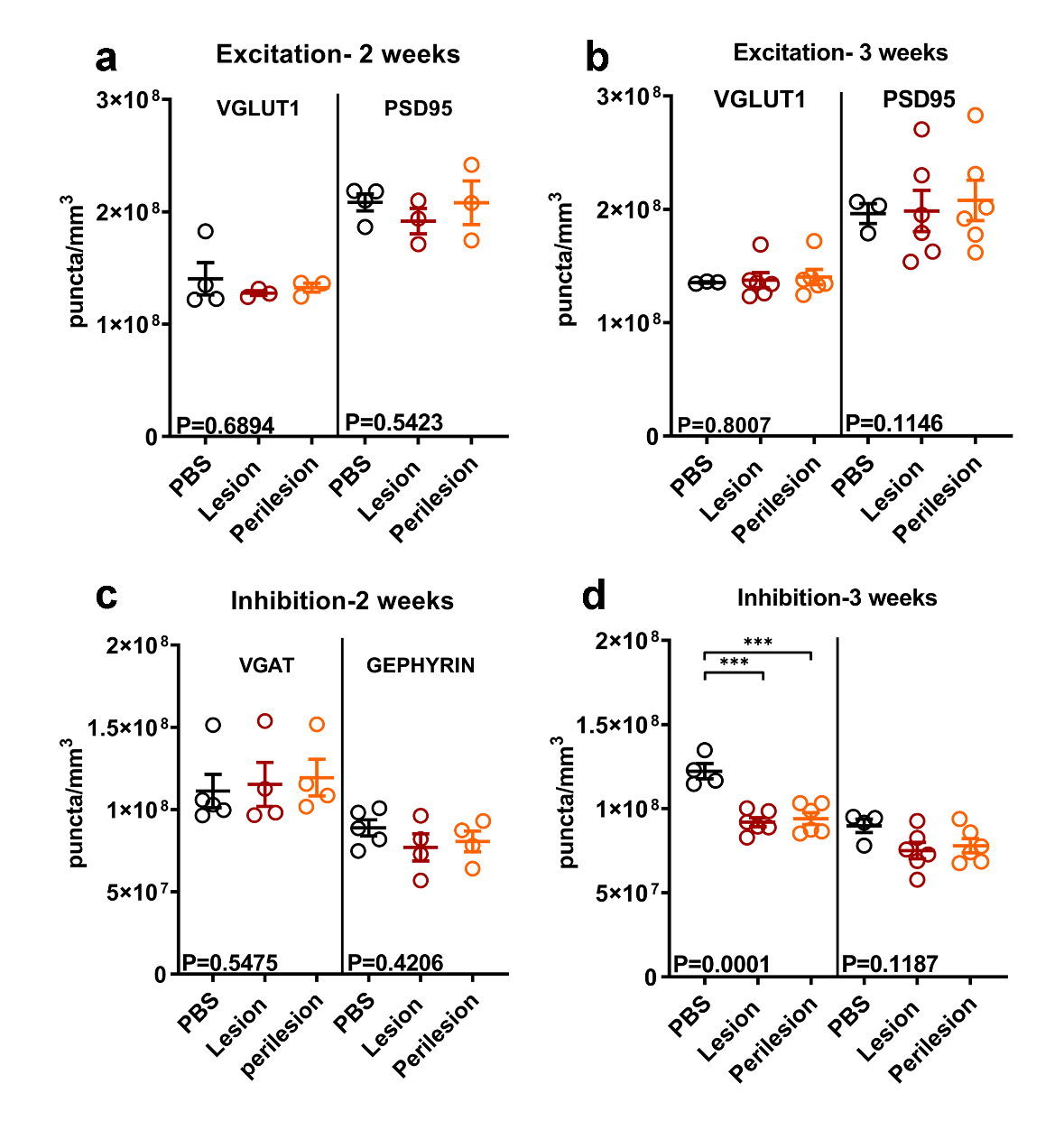


**Online Resource Fig.4. a:** Quantification of VGLUT1+ pre-synaptic or PSD95+ post-synaptic puncta in L2/3 neuropil of PBS and LPC-treated motor cortex 2 weeks post-surgery (VGLUT1+: PBS mean: 1.404e+008 ± 1.438e+007 SEM puncta/mm^3^, N=4, Lesion: mean 1.276e+008 ± 2.115e+007 SEM puncta/mm^3^, Perilesion: mean 1.325e+008 ± 4.021e+007 SEM puncta/mm^3^, N=3, each point is an animal, One-way ANOVA; PSD95+: PBS mean: 2.083e+008 ± 7.562e+007 SEM puncta/mm^3^, N=4, Lesion: mean 1.917e+008 ± 1.124e+007 SEM puncta/mm^3^, Perilesion: mean 2.081e+008 ± 3.365e+007 SEM puncta/mm^3^ N=3, each point is an animal, One-way ANOVA). **b:** Quantification of VGLUT1+ pre-synaptic or PSD95+ post-synaptic puncta in L2/3 neuropil of PBS and LPC-treated motor cortex 3 weeks post-surgery (VGLUT1+: PBS mean: 1.355e+008 ± 5.674e+007 SEM puncta/mm^3^, N=3, Lesion: mean 1.375e+008 ± 6.686e+007 SEM puncta/mm^3^, Perilesion: mean 1.403e+008 ± 6.706e+007 SEM puncta/mm^3^, N=6, each point is an animal, One-Way ANOVA; PSD95+: PBS mean: 1.962e+008 ± 8.761e+007 SEM puncta/mm^3^, N=3, Lesion: mean 1.985e+008 ± 1.812e+007 SEM puncta/mm^3^, Perilesion: mean 2.079e+008 ± 1.778e+007SEM puncta/mm^3^ N=6, each point is an animal, Kruskal-Wallis test). **c:** Quantification of VGAT+ pre-synaptic or GEPHYRIN+ post-synaptic puncta in L2/3 neuropil of PBS and LPC-treated motor cortex 2 weeks post-surgery (VGAT1+: PBS mean: 1.113e+008 ± 1.015e+007 SEM puncta/mm^3^, N=5, Lesion: mean 1.153e+008 ± 1.335e+007 SEM puncta/mm^3^, Perilesion: mean 1.194e+008 ± 1.115e+007 SEM puncta/mm^3^, N=4, each point is an animal, Kruskal-Wallis test; GEPHYRIN+: PBS mean: 8.891e+007 ± 4.877e+006 SEM puncta/mm^3^, N=5, Lesion: mean 7.708e+007 ± 8.253e+006 SEM puncta/mm^3^, Perilesion: mean 8.069e+007 ± 6.334e+006 SEM puncta/mm^3^, N=4, each point is an animal, One-way ANOVA). **d:** Quantification of VGAT+ pre-synaptic or GEPHYRIN+ post-synaptic puncta in L2/3 neuropil of PBS and LPC-treated motor cortex 3 weeks post-surgery (VGAT1+: PBS mean: 1.222e+008 ± 4.530e+007 SEM puncta/mm^3^, N=4, Lesion: mean 9.197e+007 ± 2.647e+006 SEM puncta/mm^3^, Perilesion: mean 9.407e+007 ± 3.503e+006 SEM puncta/mm^3^, N=6, each point is an animal, One-way ANOVA; GEPHYRIN+: PBS mean: 8.980e+007 ± 4.011e+006 SEM puncta/mm^3^, N=4, Lesion: mean 7.507e+007 ± 4.864e+006 SEM puncta/mm^3^, Perilesion: mean 7.794e+007 ± 4.188e+006 SEM puncta/mm^3^, N=6, each point is an animal, One-way ANOVA).

**
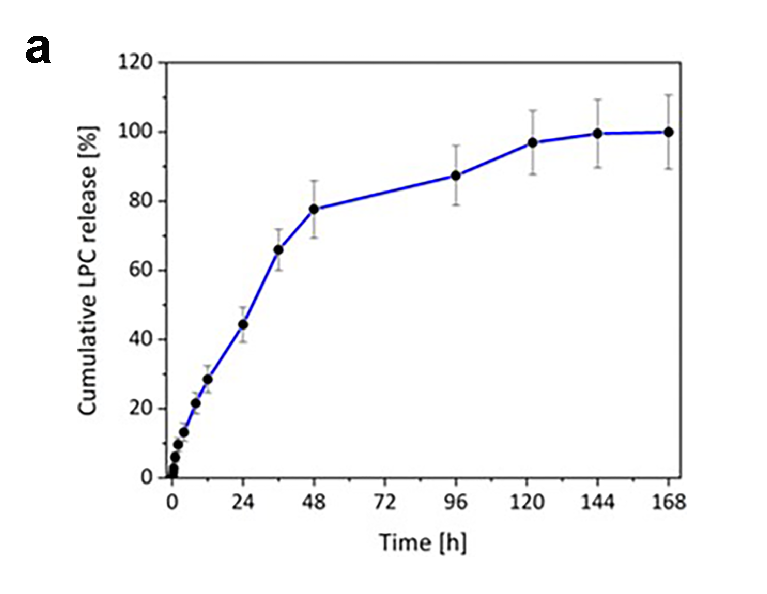
**

**Online Resource Fig. 5. a:** Release properties of LPC from the 2 mm x 0.5 mm diameter PEG cryogels. The cylindrical shaped cryogels swell in solution of LPC with the cumulative release being expressed in a percentage of the total loaded. All of the LPC is released from the cryogels over the first five days. (n = 4) (error bars represent the cumulative standard error of the mean).

**Online Resource Table 1**: Human case information (paraffin blocks).

| Sample ID | Age (years) | Sex | Cause of Death | Post-mortem interval (hours) | MS disease duration (years) |
| --- | --- | --- | --- | --- | --- |
| CO40 | 61 | F | Ovarian cancer | N/A | N/A |
| CO41 | 66 | M | Carcinoma of lung | N/A | N/A |
| CO53 | 89 | F | Hypertension, Type II diabetes, Hyperthyroidism | N/A | N/A |
| CO59 | 86 | M | Myocardial infraction | 38 | N/A |
| CO67 | 67 | F | Metastatic Ovarian Cancer | 32 | N/A |
| CO72 | 77 | M | Pneumonia, Ischaemic bowel | 26 | N/A |
| CO73 | 71 | M | Liver Cancer | 29 | N/A |
| CO74 | 84 | F | Old age | 22 | N/A |
| CO75 | 88 | M | COPD | 8 | N/A |
| CO76 | 87 | M | Pneumonia, Idiopathic pulmonary fibrosis | 31 | N/A |
| MS239 | 69 | F | Bronchopneumonia, SPMS | 42 | 51 |
| MS246 | 55 | M | Ischaemic heart disease, coronary atheroma, SPMS | 69 | 24 |
| MS285 | 61 | F | Paracetamol overdose, SPMS | 108 | 21 |
| MS290 | 66 | M | Bronchopneumonia, SPMS | 38 | 51 |
| MS309 | 74 | M | Bronchopneumonia, SPMS | 23 | 37 |
| MS344 | 57 | F | Septicaemia caused by urinary tract infection, SPMS | 14 | 15 |
| MS355 | 59 | F | Pneumonia, multiple sclerosis, SPMS | 8 | 52 |
| MS357 | 80 | F | Carcinomatosis, breast cancer, SPMS | 27 | 32 |
| MS359 | 52 | F | Pneumonia | 32 | 17 |
| MS361 | 60 | F | Advanced sigmoid colon cancer, SPMS | 10 | 34 |
| MS385 | 64 | F | Aspiration pneumonia, SPMS, atrial fibrillation | 47 | 40 |
| MS429 | 50 | M | Sepsis, intussusception, SPMS | 42 | 24 |
| MS503 | 53 | F | Bronchopneumonia, osteomyelitis, Acute pancreatitis, Gallstone obstruction, SPMS | 34 | 30 |
| MS507 | 67 | M | Chest infection, SPMS, Diabetes Mellitus | 42 | 40 |
| MS510 | 38 | F | Pneumonia, SPMS | 19 | 22 |
| MS531 | 58 | F | SPMS | 30 | 22 |
| MS549 | 50 | M | End stage SPMS | 8 | 29 |
| MS567 | 45 | F | SPMS | 48 | 23 |

**Online Resource Table 2:** Human case information for AT tissue

| Sample ID | Age (years) | Sex | Cause of Death | Post mortem Interval (hours) | MS disease duration (years) |
| --- | --- | --- | --- | --- | --- |
| BBN001.29529 | 42 | M | Suicide | 103 | N/A |
| BBN001.29531 | 42 | M | Sudden collapse while cycling | 94 | N/A |
| BBN001.29540 | 50 | M | Sudden collapse in street | 70 | N/A |
| BBN001.29533 | 57 | M | Coronary artery atheroma | 110 | N/A |
| BBN001.29693 | 49 | F | Found dead | 94 | N/A |
| BBN001.29731 | 53 | M | Suicide | 96 | N/A |
| BBN001.29906 | 51 | M | Sudden collapse | 52 | N/A |
| BBN001.30140 | 50 | M | Haemopericardium  Dissection of thoracic aorta | 122 | N/A |
| BBN001.29880 | 57 | M | Found dead in bed | 64 | N/A |
| BBN001.30169 | 48 | M |  |  | N/A |
| BBN001.32331 | 55 | M | Mesenteric ischaemia, SPMS |  | 20 |
| BBN001.32848 | 61 | F | Multi-organ failure secondary to sepsis, SPMS | 80 | 25 |
| BBN001.30890 | 70 | F | SPMS | 67 | 21 |
| BBN001.32819 | 70 | F | Mid brain stroke, Cerebrovascular disease, Type 2 diabetes, Hypertension, SPMS | 48 | NA |
| MS636 | 62 | M | SPMS | 31 | 18 |
| MS640 | 69 | F | aspiration pneumonia and SPMS | 26 | 40 |
| MS641 | 69 | F | COD bronchopneumonia and SPMS | 24 | 19 |
| MS644 | 69 | F | SPMS, bronchopneumonia, osteoporosis, | 15 | 22 |
| MS645 | 83 | F | End stage SPMS | 45 | 38 |
| MS646 | 47 | F | Aspiration, MS (RR) | 9 | 17 |
| MS658 | 69 | F | ischaemic bowel, small bowel obstruction and SPMS | 38 | 40 |
| MS665 | 74 | F | pneumonia and end stage SPMS | NA | 50 |
| MS669 | 84 | F | frailty of old age and SPMS | 12 | 59 |
| MS672 (or BBN001.35096) | 64 | F | Colon Cancer and SPMS | 44 | 31 |
| MS691 | 65 | F | Bronchopneumonia and SPMS | NA | 25 |

**Online Resource Table 3:** Table of samples used for each analysis in the study.

| Sample ID |  |  |  |  |  |  |  |  |  |  |  |
| --- | --- | --- | --- | --- | --- | --- | --- | --- | --- | --- | --- |
| MS239 | CR | SST | PV | CB | CCK |  | NEUN | Excitatory Synapses |  | SMI312 |  |
| MS246 | CR | SST | PV | CB | CCK |  | NEUN | Excitatory Synapses | Inhibitory Synapses | SMI312 |  |
| MS285 | CR | SST | PV | CB | CCK | ISLET1/2 | NEUN | Excitatory Synapses | Inhibitory Synapses | SMI312 | CUX2 |
| MS290 |  |  |  |  |  | ISLET1/2 |  |  | Inhibitory Synapses |  | CUX2 |
| MS309 | CR | SST | PV | CB | CCK | ISLET1/2 | NEUN | Excitatory Synapses | Inhibitory Synapses | SMI312 | CUX2 |
| MS344 |  |  |  |  |  | ISLET1/2 |  |  |  |  | CUX2 |
| MS355 | CR | SST | PV | CB | CCK | ISLET1/2 | NEUN | Excitatory Synapses | Inhibitory Synapses | SMI312 | CUX2 |
| MS357 | CR | SST | PV | CB | CCK | ISLET1/2 | NEUN | Excitatory Synapses | Inhibitory Synapses | SMI312 | CUX2 |
| MS359 | CR | SST | PV | CB | CCK |  | NEUN | Excitatory Synapses | Inhibitory Synapses | SMI312 | CUX2 |
| MS361 | CR | SST | PV | CB | CCK | ISLET1/2 | NEUN | Excitatory Synapses | Inhibitory Synapses | SMI312 | CUX2 |
| MS385 | CR | SST | PV | CB | CCK | ISLET1/2 | NEUN | Excitatory Synapses | Inhibitory Synapses | SMI312 | CUX2 |
| MS429 | CR | SST | PV | CB | CCK | ISLET1/2 | NEUN | Excitatory Synapses | Inhibitory Synapses | SMI312 | CUX2 |
| MS503 | CR | SST | PV | CB | CCK | ISLET1/2 | NEUN | Excitatory Synapses | Inhibitory Synapses | SMI312 | CUX2 |
| MS507 | CR | SST | PV | CB | CCK | ISLET1/2 | NEUN | Excitatory Synapses | Inhibitory Synapses | SMI312 | CUX2 |
| MS510 | CR | SST | PV | CB | CCK | ISLET1/2 | NEUN | Excitatory Synapses | Inhibitory Synapses | SMI312 | CUX2 |
| MS531 | CR | SST | PV | CB | CCK | ISLET1/2 | NEUN | Excitatory Synapses | Inhibitory Synapses | SMI312 | CUX2 |
| MS549 | CR | SST | PV | CB | CCK | ISLET1/2 | NEUN | Excitatory Synapses | Inhibitory Synapses | SMI312 | CUX2 |
| MS567 |  |  |  |  |  | ISLET1/2 |  |  |  |  |  |
| CO40 | CR | SST | PV | CB | CCK | ISLET1/2 | NEUN | Excitatory Synapses | Inhibitory Synapses | SMI312 | CUX2 |
| CO41 | CR | SST | PV | CB | CCK | ISLET1/2 | NEUN | Excitatory Synapses | Inhibitory Synapses | SMI312 | CUX2 |
| CO53 | CR | SST | PV | CB | CCK | ISLET1/2 | NEUN | Excitatory Synapses | Inhibitory Synapses | SMI312 | CUX2 |
| CO59 | CR | SST | PV | CB | CCK | ISLET1/2 | NEUN | Excitatory Synapses | Inhibitory Synapses | SMI312 | CUX2 |
| CO67 | CR | SST | PV | CB | CCK | ISLET1/2 | NEUN | Excitatory Synapses | Inhibitory Synapses | SMI312 | CUX2 |
| CO72 | CR | SST | PV | CB | CCK | ISLET1/2 | NEUN | Excitatory Synapses | Inhibitory Synapses | SMI312 | CUX2 |
| CO73 | CR | SST | PV | CB | CCK | ISLET1/2 | NEUN | Excitatory Synapses | Inhibitory Synapses | SMI312 | CUX2 |
| CO74 | CR | SST | PV | CB | CCK | ISLET1/2 | NEUN | Excitatory Synapses | Inhibitory Synapses | SMI312 | CUX2 |
| CO75 | CR | SST | PV | CB | CCK | ISLET1/2 | NEUN | Excitatory Synapses | Inhibitory Synapses | SMI312 | CUX2 |
| CO76 | CR | SST | PV | CB | CCK | ISLET1/2 | NEUN | Excitatory Synapses | Inhibitory Synapses | SMI312 | CUX2 |

**Online Resource Table 4:** Antibody information

| Antibodies | Source | Identifier | Dilution |
| --- | --- | --- | --- |
| **Monoclonal IgG2b anti-Islet-1&Islet-2 homeobox** | Developmental Studies Hybridoma Bank | Cat# 39.4D5, RRID:AB_2314683 | 1/50 |
| **Polyclonal anti-CUX2** | Stratech Scientific Ltd | Cat# BS-11832R-BSS, RRID:N/A | 1/200 |
| **Polyclonal anti-parvalbumin** | Swant | Cat# PV27, RRID:AB_2631173 | 1/1000 |
| **Monoclonal IgG1 anti-Neurofilament Marker (pan axonal, cocktail)** | Biolegend | Clone SMI 312, Cat# 837904, RRID:AB_2566782 | 1/100 |
| **Monoclonal IgG2b anti-Somatostatin** | MERCK-Millipore | clone YC7, Cat# MAB354, RRID:AB_2255365 | 1/250 |
| **Monoclonal anti-Calretinin (CR)** | Swant | Cat# 6B3, RRID:AB_10000320 | 1/100 |
| **Polyclonal anti-Cholecystokinin** | Abcam | ab134713 RRID: N/A | 1/100 |
| **monoclonal IgG1 anti Calbindin D-28k antibody** | Swant | Cat# 300, RRID:AB_10000347 | 1/100 |
| **Polyclonal Anti-Synapsin I** | MERCK-Millipore | Cat# AB1543P, RRID:AB_90757 | 1/250 |
| **Monoclonal IgG2a, anti-PSD-95 MAGUK scaffolding protein** | UC Davis/NIH NeuroMab Facility | Clone: k28/43, Cat# 75-028, RRID:AB_2292909 | 1/250 |
| **Polyclonal anti-Vesicular Glutamate Transporter 1** | MERCK-Millipore | Cat# AB5905, RRID:AB_2301751 | 1/250 |
| **Polyclonal anti-Vesicular Glutamate Transporter 2** | MERCK-Millipore | Cat# AB2251-I, RRID:AB_2665454 | 1/250 |
| **Polyclonal anti-** **Anti-VGAT (GABA transporter in the membrane of synaptic vesicles)** | Synaptic Systems | Cat# 131 004, RRID:AB_887873 | 1/250 |
| **Monoclonal IgG1 anti-gephyrin** | Synaptic Systems | Clone: mAb7a, Cat# 147 011C3, RRID:AB_887716 | 1/100 |
| **Monoclonal IgG2a anti- MBP (aa82-87)** | Bio-Rad | Cat# MCA409S, RRID:AB_325004 | 1/300 |
| **Monoclonal IgG anti Iba1** | Abcam | Clone: EPR16588, Cat# ab178846, RRID:AB_2636859 | 1/500 |
| **Polyclonal anti- Glial Fibrillary Acidic Protein** | Agilent | Cat# Z0334, RRID:AB_10013382 | 1/500 |
| **Polyclonal anti-CD3** | abcam | Cat# ab5690, RRID:AB_305055 | 1/100 |
